# Heart Rate Variability Reflects the Natural History of Physiological Development in Healthy Children and Is Not Associated with Quality of Life

**DOI:** 10.1371/journal.pone.0091036

**Published:** 2014-03-13

**Authors:** Georg Seifert, Gabriele Calaminus, Andreas Wiener, Dirk Cysarz

**Affiliations:** 1 Charité–Universitätsmedizin Berlin, Department of Paediatric Oncology and Haematology, Berlin, Germany; 2 University Hospital Muenster, Childrens Hospital, Department for Paediatric Haematology and Oncology, Münster, Germany; 3 Integrated Curriculum for Anthroposophic Medicine, Institute of Integrative Medicine, University of Witten/Herdecke, Witten, Germany; University of Adelaide, Australia

## Abstract

**Background:**

Quality of life (QoL), being the sum expression of diverse influencing factors, is not easy to determine. A clinically relevant option would be to identify and measure quality of life on the basis of physiological parameters which correlate plausibly and statistically with psychometrically measured QoL. Analysis of heart rate variability (HRV) offers readily measurable physiological parameters which could be of use here. A correlation of HRV with both course of disease and QoL has been reported in patients with chronic illness. Various psychometric instruments have been developed for use in paediatric oncology. The aim of this study was to obtain data on HRV and QoL and their correlations, initially in healthy children.

**Methods:**

Holter ECG and quality of life were examined in 160 children and adolescents (72 male) aged between 8 and 18 years. QoL was determined with the established questionnaire PEDQoL. Standard parameters of HRV from the frequency domain were calculated and correlated with QoL domains using Spearman (nonparametric) correlation analysis.

**Results:**

Minor but significant associations were revealed only with regard to the PEDQoL domain “autonomy” on the one hand and heart rate and HRV (e.g. MRR, MRRn, MRRd, HRV_ULF, SDNN) parameters which evidently reflect distinct physiological functions on the other.

**Conclusions:**

In healthy children and adolescents we have a first indication that there is a correlation between parameters of HRV and QoL. However, to a greater extent, HRV reflects associated physiological processes of the autonomic nervous system. A higher correlation is more likely to be found in chronically ill children.

## Introduction

Quality of life (QoL) in children and adolescents is the sum expression of diverse physical, emotional and mental processes and their integrative processing and experiencing by the individual. Endogenous and exogenous physiological or pathological processes influence its perception [1]. Many illnesses are associated with reduced QoL [2]–[4]. Although there are now numerous instruments available for quality of life measurement in paediatric medicine, often the interpretation of individual results is not easy because of the non-linear relation between a subjective rating and an assessment by others in situations of health state changes [5]–[8]. Corresponding individual observations are sometimes referred to as “*paradox of satisfaction/dilemma of dissatisfaction*” [9] or “*response shift phenomenon*” in case of repeated measurements. Response shift “refers to the changes in internal standards, in values, or in the conceptualization of QOL which are catalyzed by health state” [10], [11].

A further problem is that measurement of QoL is more difficult the younger the children are. Therefore the investigation of more “objective” measures in order to complete measurement and to increase interpretability, e.g. in a clinical context, is becoming increasingly important particularly in children with chronic disease, in paediatric oncology for example [12], [13].

One promising way to come to a better understanding of individual QoL ratings (e.g. in the course of a medical treatment) is to identify complementary parameters reflecting a physiological process which could plausibly constitute a kind of salutogenic marker of QoL, i.e. which have a relevant influence on health-related quality of life. From the physiological perspective, functional processes related to the heart would appear to be a good starting point. Here we would be looking particularly at parameters of heart rate variability (HRV) analysis which can reflect states such as stress, tension, relaxation, sleep and physical activity [14], [15].

Standard parameters of HRV bear a physiologically possible and plausible connection with physical exercise, activity, tension, relaxation and physical fitness/activity. Many parameters in the time domain (mean normal-to-normal interval – mNN, standard deviation of normal-to-normal intervals – SDNN) and in the frequency domain (ultra low frequency oscillations – ULF, very low frequency oscillations – VLF, low frequency oscillations – LF, high frequency oscillations – HF) contain information that can be related to the functioning of the autonomic nervous system (ANS) [16]. HF and SDNN are related to parasympathetic modulations whereas LF is related to both, sympathetic and parasympathetic modulations. Hence, the parameter LF/HF can be cautiously interpreted as ‘sympathovagal balance’. However, the physiological interpretation of VLF and ULF still needs to be clarified. As shown recently, the parameters in the time and frequency domain show a clear development in the course of childhood, puberty and into young adulthood. This development depends on age, gender and day/night rhythm [17]. The variations with age show that this physiological development is not linear (parameters only increase or decrease, respectively) but some HRV parameters reflecting non-linear properties show a local maximum at the age of 9 years. Hence, they are associated with certain milestones of physical development during childhood and adolescence. For example, notable changes occur during prepuberty and puberty, possibly reflecting hormonal changes [18]–[21].

Interestingly, when we speak of feeling depressed or in good spirits we often use expressions such as “heavy-hearted” or “light-hearted”. From the physiological point of view there is an element of truth in these expressions as patients suffering from depression, for example, display a significant and measurable reduction in HRV which is reversible in the case of successful treatment [22]–[24]. Numerous studies have shown that cardiac rhythm – specifically HRV, for example – is modulated by a large number of different emotional [25], [26], neuronal [27]–[29] and humoral factors [30]–[32]. In the clinical sector there are numerous data showing a correlation between HRV parameters and the course of various diseases in adults, occasionally also in combination with data on QoL [22], [33]–[39].

It appears to be the case that HRV can show very subtle functional changes which indicate a current health impairment or beginning pathology which is not yet directly visible clinically.

For diseases such as heart attack [40] or depression [41] a deterioration of HRV parameters has been described which can stabilise or improve again if the disease shows a favourable course [40]. In parallel, the course of disease may also correlate with measured quality of life.

Both quality of life and HRV are influenced by physical activity [14]. Restorative sleep, physical activity and stress can influence HRV parameters and quality of life [42]–[47]. As further development of the instruments for measurement of quality of life or the development of complementary methods is of great interest, it appears appropriate to examine whether certain domains of QoL which are believed to be particularly influenced by physical activity, correlate with apparently corresponding sensitive parameters of HRV. These are, in particular, the physiologically plausible parameters of the frequency spectrum and movement dependent parameters. In order to advance the understanding of individual QoL rating and with a view to studies in chronically ill children, the purpose of the present study was to examine our assumption that elements of a QoL instrument frequently used in paediatric oncology correlate substantially with parameters of HRV.

We analyzed the association between QoL parameters and physiological parameters of HRV in a sample of 160 children out of a total sample of 469 healthy children and adolescents [17]. As further lifestyle markers we also analyzed the time spent in front of a television or computer and the body mass index (BMI) as these are parameters of physical activity and thus also of physiological fitness.

## Methods

### Subjects

A total of 469 children and adolescents were initially enrolled in this cross-sectional study (see Table 1). Of these, 160 subjects (age range 8 to 18 years, 88 females, 72 males) had an ECG recording suitable for further analysis and completed the quality of life questionnaires. Note that the questionnaires were only designed for children aged 8 years and above and, hence, only children in this age group were included in the analysis. None of the subjects had any history of cardiovascular disease or diabetes. The children were born and educated in Germany and spoke fluent German. To obtain an average school sample we did not exclude subjects with chronic diseases. We are aware that diseases such as bronchial asthma (N = 2), chronic obstructive pulmonary disease (N = 1) or migraine (N = 1) may influence cardiovascular parameters. On the other hand, allergies (N = 3), atopic eczema (N = 1) and noctunuresis (N = 1) do not alter cardiovascular parameters. We also noted that 5 subjects were taking medication for an attention-deficit hyperactivity disorder and 4 subjects were taking naturopathic medication. It has not yet been investigated whether these medications have a systematic impact on cardiovascular functions.

**Table 1:** From a group of 469 children (409 children with ECGs) both ECGs and evaluable questionnaires on quality of life were available for n = 160 (≥8 years).

	Mean	SD	Median	Min/Max
Age	12.06	3.36	11	8/18
BMI (n = 137)	20.34	4.05	19.65	14.2/34.94
Sex	72 male (45%) 88 female (55%)			
PC [hours]	1.11	0.943	1	0/3
Cell phone [hours]	0.92	0.832	1	0/3

The table shows the distribution parameters of age, sex, BMI and use of PC/cell phone.

Written informed consent was obtained from the guardians and also the subjects, if applicable, in accordance with the Declaration of Helsinki. The study protocol had been approved by the local ethics committee of Charité-Universitätsmedizin Berlin.

### ECG Recordings

The 24 h-Holter ECGs were recorded under everyday conditions. The digital Holter device (Medikorder MK3, Schiller Engineering, Graz, Austria) had an internal sampling rate of 4096 Hz and the R-peaks were automatically detected by the device with a precision of <1 ms. The ECGs (at a sampling rate of 256 Hz) as well as the automatically identified times of the R-peaks were stored on a personal computer. In the case of artefacts and premature beats the timings of the respective R-peaks were changed according to the saved ECGs (<1.5% of all recorded R-peaks). Ventricular ectopic beats were omitted from the analysis because they did not originate in the sinus node (<0.1% of all beats). The RR-interval series was created by calculating the temporal difference between successive R-peaks. This series was the basis for further calculations.

### Heart Rate Variability

The variability of the RR-interval series contains valuable information which may partly be interpreted in terms of the modulations of the two branches of the ANS. In the time domain the mean of normal-to-normal R-R intervals (mRR) and the corresponding standard deviation (SDNN) were calculated. In the frequency domain the extent of very low frequency oscillations (VLF: 0.0033 to 0.04 Hz), low frequency oscillations (LF: 0.04 to 0.15 Hz) and high frequency oscillations (HF: 0.15 to 0.4 Hz) were quantified using the fast Fourier transformation [16]. The measures were calculated for each consecutive 1-hour epoch of the recording. Subsequently they were averaged to obtain 24 h averages. Daytime and nighttime values were obtained by calculating the average values from midnight to 6 am (nighttime) and from 9 am to 6 pm (daytime). This differentiation is useful to obtain estimates for the functioning of the ANS during activity (daytime values) and recovery (nighttime values) as well as a 24 h average. Furthermore, the ultra low frequency oscillations (ULF: ≤0.0033 Hz) and VLF oscillations were quantified using the R-R interval series from the entire recording.

HF and LF may be interpreted in terms of modulations of the ANS. HF and SDNN are associated with modulations of the parasympathetic branch of the ANS whereas LF is associated with modulations of both the sympathetic and parasympathetic branches [16], [48]. The interpretation of VLF and ULF is still a matter of debate as many different influences contribute to the power in these frequency ranges [48]. Recently, ULF has been suggested to reflect variations of physical activity [49]. HF and LF can also be used to indicate the level of stress because emotional and physical stress decrease parasympathetic control or increase sympathetic modulations, or both [50]. Generally, HRV is reduced during stress and, hence, the other measures are also reduced.

We also calculated measures to assess non-linear properties of HRV. These measures provide information that cannot be derived from the linear parameters. In particular, fractal scaling properties were quantified using the slope of the spectral density in the range 0.0001 to 0.01 Hz (large scale properties, comprising up to 10000 beats), detrended fluctuation analysis (DFA) on medium (ALPHA2, 12 to 1000 beats) and short scales (ALPHA1, 4 to 11 beats). ALPHA1, ALPHA2 and the slope beta quantify self-similarity on short, intermediate amd long time scales. Decreased values of ALPHA1 and ALPHA2 correspond to worse outcome, e.g. after myocardial infarction [51]. Complexity was assessed using Approximate Entropy (ApEn) as a measure of regularity of a time series [52]. Higher values correspond to higher irregularity. Further details have been published by Cysarz et al. [17].

In the following, the parameters with the appendix ‘24’ represent values calculated for the entire recording, the appendix ‘d’ indicates daytime values (9 am to 6 pm) and the appendix ‘n’ indicate nighttime values (0 to 6 am).

### QoL Assessment

HRQoL was estimated by self-rating with a validated PEDQoL questionnaire [53], [54] for children and adolescents with cancer including the domains physical function (K), emotional functioning (E), cognition (C), autonomy (Aut), social functioning family (Fam), social functioning friends (Fr) and body image (KB).

The questionnaire contains 48 items which are distributed among 6 domains: physical functioning and pain (9 items), emotional functioning (6 items), cognition (6 items), autonomy (6 items), social functioning family (6 items), social functioning friends (6 items), and body image (9 items), as well as general well-being.

Questions about physical functioning ask how the children rate their physical abilities, e.g. when compared to friends. In the domain emotional functioning the patients were asked about the mood they were in, if they felt happy or sad. The domain cognition included questions about the children’s ability to study, to do their homework, also in comparison to their classmates. Questions regarding autonomy asked how the children saw their ability to decide or do things on their own. The domain social functioning family asked about their relation to their family/parents. The items in the domain social functioning friends asked the children about their relation to peers. Questions regarding body image included how satisfied they were with their appearance.

The PEDQoL questionnaire consists of four-level Likert items. The format is “always”, “often”, “rarely”, and “never”.

All children were asked to answer the questionnaire on their own. Their parents were not in the same room during this procedure. In the case of comprehension problems the children were asked to mark the question and additional explanation was given by the study coordinator after they had completed the questionnaire. Media consumption was measured by a questionnaire (never; <1 hour/day 1–2 hours/day; >2 hours/day.

### Statistics

#### HRV parameters

The results are presented as mean ± STD. The spectral parameters had to be log-transformed to yield normal distributions. All other measures were normally distributed.

#### QoL parameters and correlation analysis

For analysis the PEDQoL domains were evaluated separately. The four verbal markers of the rating scale were positioned on a hypothetical QoL scale ranging from 0% (worst QoL) to 100% (best possible QoL) and distinct percentage values were assigned. The respective attributions were 25% (“never”), 50% (“rarely”), 75% (“often”), and 100% (“always”). For each PEDQoL domain the respective percentages were added together and the mean percentage score was calculated. Finally, the scores of each patient were merged and the sample-based median percentage score for each single domain was calculated.

Data were tested for Gaussian distribution using the Shapiro-Wilk test for normality. To test correlation between QoL and EKG parameters for the various variables, Pearson’s and Spearman’s correlation coefficients were calculated respectively. A value p<0.05 was considered statistically significant. A one-tailed p-value was used because the hypothesis was restricted to one direction.

For the sake of clarity only those variables which were considered relevant for discussion are presented. Since this investigation is of exploratory nature we refrained from carrying out any multivariate analyses.

## Results

Table 1 shows the basic characteristics of the study sample. QoL data and Holter EGGs were available for 160 subjects (age ≥8 years, 72 male, 88 female). The average BMI was 20.37 indicating normal weight on average (http://apps.who.int/bmi/index.jsp). Average times spent using a personal computer (PC) or a cell phone were one hour each.

### Heart Rate Variability

**Figure 1:**
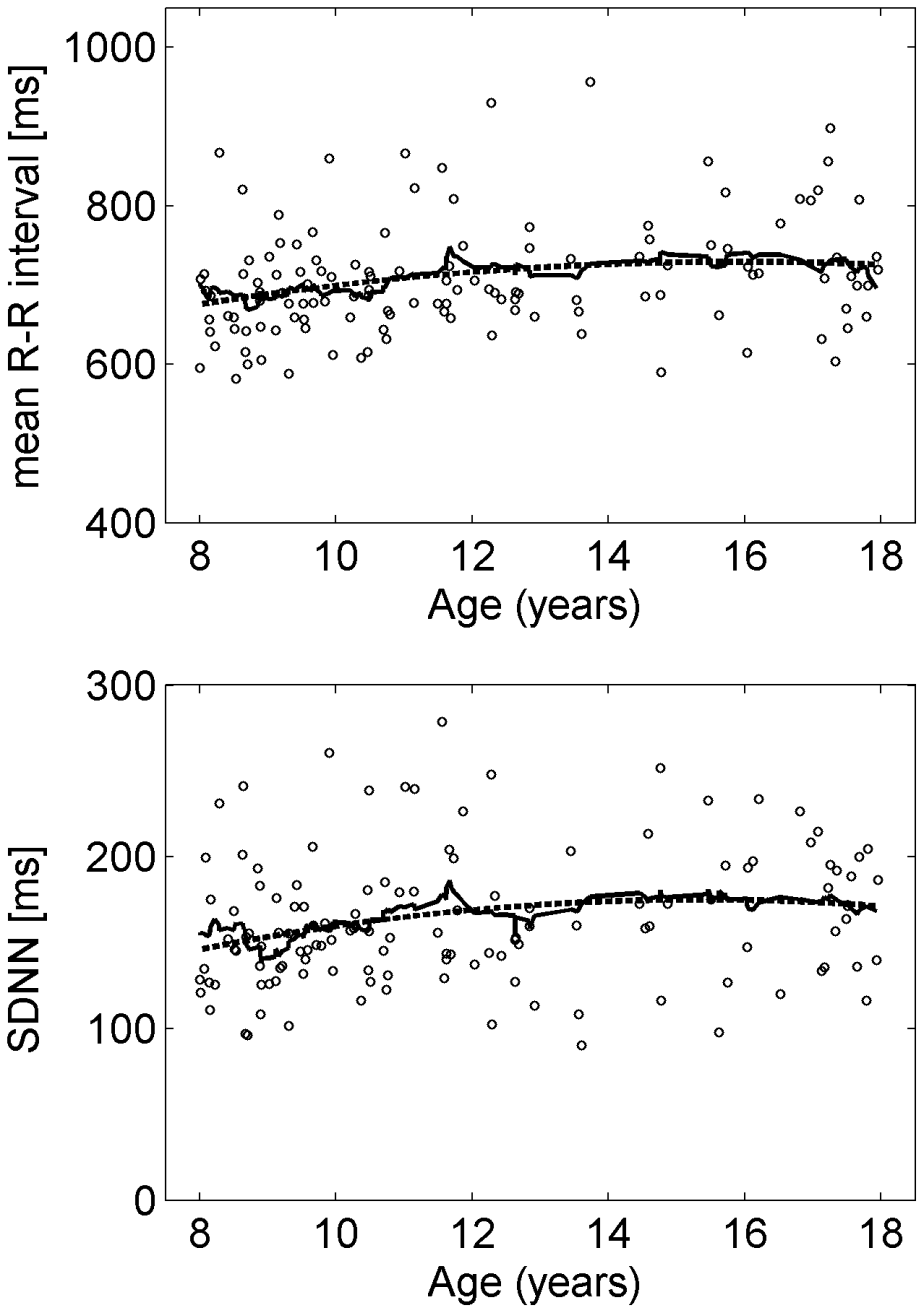
Examples of variations of HRV parameters with age. Age-related variations of the average RR interval (top) and the standard deviation of the distance between normal-to-normal heartbeats (bottom). The lines denote a polynomial fit of order 2 (dashed line) and the moving average (full line).

As an example of the age related variations of HRV the time domain parameters mNN and SDNN are shown in Figure 1. As age-related variations of the parameters are less apparent than in the whole group (ages ranging from 2 to 21 years) [17] the results are summarized for the whole group. Table 2 lists the averages and standard deviations of the HRV parameters. All parameters showed differences between daytime and night-time values.

**Table 2:** HRV parameters (mean ± sd) of the whole group calculated for the entire recording (24 h), daytime and nighttime.

	24 h	Day	Night
Mean RR [ms]	708±75	617±69	891±122
SDNN [ms]	164±41	99±27	116±35
ULF [ln ms2]	9,8±0.5	–	–
VLF [ln ms2]	7.9±0.5	7.6±0.5	8.2±0.6
LF [ln ms2]	7.4±0.5	7.1±0.5	7.6±0.6
HF [ln ms2]	7.2±0.8	6.4±0.8	7.7±0.9
Slope beta	−1.09±0.14	–	–
Alpha 2	0.99±0.05	1.01±0.06	0.93±0.07
Alpha 1	1.14±0.16	1.27±0.17	0.92±0.18
ApEn	0.82±0.24	0.66±0.24	1.14±0.34

Note that the parameter ‘slope beta’ can only be calculated for the entire recording.

### Correlation of HRV Parameters, QoL, Media Consumption and BMI

The results of the different PEDQoL domains are shown in Table 3. In all domains the measured scores correspond to those of the reference group for standardisation of the PedQoL. It can therefore be assumed that our sample is a representative group of healthy children and adolescents.

**Table 3:** Distribution parameters of QoL domains.

	Median	Min/Max
K	37,5	25/87,5
EV	40	25/80
C	45	25/90
AUT	45,83	25/79,2
Fr	40	25/100
Fam	37,5	25/100
KB	45	25/95

Physical function (K), emotional functioning (E), cognition (C), autonomy (Aut), social functioning family (Fam), social functioning friends (Fr) and body image (KB).

Generally, there are only few correlations between HRV parameters (e.g. MRR and SDNN) and PEDQoL domains. Taking the whole group such correlations can only be found for the parameter ‘autonomy’ (see Table 4). Parameters in the time domain (MRR24, MRRn, MRRd, SDNN24) in the frequency domain (HF24, LFd, VLFd, ULF24) and fractal scaling like properties (ALPHA1_24, ALPHA1d) show correlations. These correlations are weak (i.e., all correlation coefficients <0.3) and negative, i.e., the higher the domain ‘autonomy’ the lower the HRV parameters. E.g. the higher the domain ‘autonomy’ the lower the mean RR-interval of the entire recording (MRR24), i.e. the high the average heart rate.

**Table 4:** Correlations between HRV parameters and the PEDQOL domain “autonomy” taking into account the whole group of children and adolescents.

	AUT
MRR24	−0,279**
MRRn	−0,281**
MRRd	−0,248**
SDNNd	−0,158*
SDNN24	−0,192**
HLF24	−0,141*
VLFd	−0,144*
ULF24	−0,242**
LFd	−0,143*
Alpha1_24	−0,229**
Alpha1d	−0,221**

**<0.01 one-tailed; *<0.05 one-tailed.

We also calculated the correlations for the age groups ≤11 years (N = 82) and >11 years (N = 72). Again, most correlations were found with respect to the PEDQoL domain ‘autonomy’ (see Table 5). In the age group ≤11 years most correlations were found with respect to the standard HRV parameters (MRR24, MRRn, SDNN24, ULF24, VLF24, VLFn) and with respect to complexities (ApEn24). These correlations were also weak and negative. The PEDQoL domain ‘physical function’ did not correlate to any HRV parameter in this age group. Interestingly, in the age group>11 years only HRV parameters representing fractal scaling like properties (Alpha1n, Alpha1d) showed negative correlations to the PEDQoL domain ‘autonomy’. In this age group also a fractal scaling like property (Alpha1d) showed a negative correlation to the domain ‘physical function’. Note that these correlations were also weak.

**Table 5:** Correlations between HRV parameters and the PEDQOL domains “autonomy (AUT)” and “physical function (K)” for the age groups ≤11 years (N = 82) and >11 years (N = 78).

	Age ≤11 years		Age >11 years	
	AUT	K	AUT	K
MRR24	−0,303**	n.s.	n.s.	n.s.
MRRn	−0,257**	n.s.	n.s.	n.s.
SDNN24	−0,215*	n.s.	n.s.	n.s.
ULF24	−0,231*	n.s.	n.s.	n.s.
VLF24	−0,293**	n.s.	n.s.	n.s.
VLFn	−0,271**	n.s.	n.s.	n.s.
Alpha1n	n.s.	n.s.	−0,207*	n.s.
Alpha1d	n.s.	n.s.	−0,314**	−0,203*
ApEn24	−0,208*	n.s.	n.s.	n.s.

**<0.01 one-tailed; *<0.05 one-tailed.

Gender differences were also observed (see Table 6). The female group and the male group both showed correlations with the PEDQoL domain ‘autonomy’ in a variety of parameters (MRR24, MRRn, MRRd, ULF24, Alpha1d, Alpha2d). The female subjects also showed correlations with the PEDQoL domain ‘autonomy’ for the parameters SDNNd and ULF24 whereas the male subjects also showed correlations with the PEDQoL domain ‘autonomy’ for the parameters SDNN24, VLFn, VLFd, LFd, Alpha2_24 and Alpha2n. Again, all correlations were weak and negative.

**Table 6:** Correlations between HRV parameters and the PEDQOL domains “autonomy (AUT)” and “physical function (K)” for the female (N = 88) and male (N = 72) children and adolescents.

	female		male	
	AUT	K	AUT	K
MRR24	−,298**	n.s.	−,339**	n.s.
MRRn	−,249**	n.s.	−,379**	n.s.
MRRd	−,328**	n.s.	−,258**	
SDNN24	n.s.	n.s.	−,253**	n.s.
SDNNd	−,229**	n.s.	n.s.	n.s.
ULF24	−,167*	n.s.	−,326**	n.s.
VLFn	n.s.	n.s.	−,182*	n.s.
VLFd	n.s.	n.s.	−,219*	n.s.
LFd	n.s.	n.s.	−,186*	n.s.
Alpha1n	−,186*	n.s.	−,195*	n.s.
Alpha1d	−,177*	n.s.	−,359**	n.s.
Alpha2_24	n.s.	n.s.	−,293**	n.s.
Alpha2n	n.s.	n.s.	−,266**	n.s.

**<0.01 one-tailed; *<0.05 one-tailed.

The PEDQoL domains “autonomy” and “physical function” both correlated negatively with the amount of computer use and playing computer games (see Table 7). The correlation between usage of computer and ‘autonomy’ was slightly stronger than the other correlations (r = −0,471). Hence, the more using the computer (and playing computer games) the less the autonomy. A similar (but weaker) relationship was found with respect to the amount of usage of cell phones.

**Table 7:** Correlations between HRV parameters and the PEDQOL domain “autonomy”; PC: Computer/Games.

	AUT	K
PC [hours]	−0,471**	−0,213**
Cell phone [hours]	−0,297**	n.s.

**<0.01 one-tailed; *<0.05 one-tailed.

## Discussion

If our assumption that HRV parameters reflecting different aspects of the ANS can be used for objectively measuring QoL were correct, this could potentially provide an interesting method to supplement the existing psychometric test procedures. In particular the HRV parameters which are associated with tension, stress, relaxation and physical activity [14], could represent a functional physiological correlate for certain areas of QoL. In chronically ill adult patients a connection between HRV parameters and the course of certain diseases has been shown [23], [29], [55]–[57]. With a view to examining whether this also applies to chronically ill children, we began by looking for correlations in healthy children. Standard parameters of HRV were calculated and correlated with QoL data.

However, the data presented showed only a weak correlation between QoL and HRV which are not greater than 0.38 and thus not sufficiently strong to suggest relevant connections. Interestingly, most correlations were found in the age group ≤11 years whereas the age group>11 years shows correlations in considerably fewer parameters. Hence, it seems that especially younger children contributed to the results of the entire group. Furthermore, female subjects showed more correlations than male subjects. Hence, also male subjects may have contributed stronger to the results in the whole group than the female subjects.

Looking at subjects with increased media consumption, however, we find an overall picture to some degree plausible which shows already known associations [53], namely that children and adolescents who consume media such as television, computers and computer games frequently and for long periods have considerably less exercise and are altogether more inactive [58]. This has a confirmed influence on body weight development [59]. A raised BMI in turn has a direct influence on the cardiovascular system and HRV [60] and is associated, among other things, with an increased heart rate and autonomic function [61] and cardiovascular risks. An increased heart rate results in a decrease in heart rate-dependent HRV parameters [16]. It is plausible and in agreement with the literature that children in this group also have a lower-perceived quality of life [62]. Increased media consumption can compromise family interaction and probably results in more frequent negative perception of body image, motor development and autonomy. In reverse there is a negative/positive correlation between sport, autonomy and individual HRV parameters. These findings show that our data can be meaningfully interpreted and correspond to already known associations. Overall, however, these areas only show a weak correlation. This means that small physiologically plausible relationships exist but that their influence on HRV is only minor. Gender and age have a considerably greater influence [17]. In this context the results are limited by the fact that we were not able to perform separate analyses for girls and boys as the sample size was too low. However, as we have shown gender undoubtedly influences HRV [17]. Hence, the main finding of the study is that in healthy children HRV essentially reflects functional physiological aspects which follow a set pattern of development rather than being influenced by individual domains of QoL. HRV is thus a relatively independent marker of autonomic regulation in healthy children and adolescents with normal body weight and little influenced by social and emotional factors. However, this does not mean that in the case of chronic disease pathological associations cannot also occur in children. It should be noted that establishing a clear relationship between HRV and QoL is per se difficult because of large interindividual variation of HRV parameters [17]. Many factors present during measurement affect HRV, as well as factors in the past such as pre-natal development [63].

Pathological conditions could be associated with a measurable deterioration in autonomic regulation and at the same time also cause reduced QoL, as is already known for adult patients [64]–[66]. In the case of sick adults the available data on the association between QoL, course of disease and HRV show a correlation. Here impairment of HRV correlates clearly with the course of disease in patients with heart disease as well as in patients with many other chronic diseases. It can be assumed that pathological processes can have a marked effect on the heart itself and on autonomic regulation as well as on the entire neuronal and humoral system, as a result of which their effects are reflected in changes in HRV [31], [67]–[70]. For children comparable investigations have yet to be conducted.

## Conclusion

In healthy children and adolescents QoL displays low correlation with heart rate and parameters of HRV. Although this exploratory study showed moderate correlations we conclude that in healthy children and adolescents, HRV essentially reflects relatively independent functional markers of the autonomic regulation of the cardiovascular system. However, further studies are necessary to examine whether there is a correlation between QoL and HRV in chronically ill children. In healthy children, HRV essentially reflects the intact autonomic nervous system and the natural history of physiological development.

## References

[1] Payot A, Barrington KJ (2011) The quality of life of young children and infants with chronic medical problems: review of the literature. Curr Probl Pediatr Adolesc Health Care 41(4): 91–101. 21440223 10.1016/j.cppeds.2010.10.008

[2] Nordlund B, Konradsen JR, Pedroletti C, Kull I, Hedlin G (2011) The clinical benefit of evaluating health-related-quality-of-life in children with problematic severe asthma. Acta Paediatr 2011(19): 1651–2227. 10.1111/j.1651-2227.2011.02359.x 21595747

[3] Taylor J, Jacoby A, Baker GA, Marson AG (2011) Self-reported and parent-reported quality of life of children and adolescents with new-onset epilepsy. Epilepsia 2011(13): 1528–1167. 10.1111/j.1528-1167.2011.03094.x 21569020

[4] van Litsenburg RR, Huisman J, Hoogerbrugge PM, Egeler RM, Kaspers GJ, et al (2011) Impaired sleep affects quality of life in children during maintenance treatment for acute lymphoblastic leukemia: an exploratory study. Health Qual Life Outcomes 9: 25. 21496357 10.1186/1477-7525-9-25 PMC3095992

[5] Solans M, Pane S, Estrada MD, Serra-Sutton V, Berra S, et al (2011) Health-related quality of life measurement in children and adolescents: a systematic review of generic and disease-specific instruments. Value Health 11(4): 742–764. 10.1111/j.1524-4733.2007.00293.x 18179668

[6] De Civita M, Regier D, Alamgir AH, Anis AH, Fitzgerald MJ, et al (2005) Evaluating health-related quality-of-life studies in paediatric populations: some conceptual, methodological and developmental considerations and recent applications. Pharmacoeconomics 23(7): 659–685. 15987225 10.2165/00019053-200523070-00003

[7] Frisen A (2007) Measuring health-related quality of life in adolescence. Acta Paediatr 96(7): 963–968. 17498184 10.1111/j.1651-2227.2007.00333.x

[8] Eiser C, Jenney M (2007) Measuring quality of life. Arch Dis Child 92(4): 348–350. 17376942 10.1136/adc.2005.086405 PMC2083670

[9] Pemberger S, Jagsch R, Frey E, Felder-Puig R, Gadner H, et al (2005) Quality of life in long-term childhood cancer survivors and the relation of late effects and subjective well-being. Support Care Cancer 13(1): 49–56. 15565275 10.1007/s00520-004-0724-0

[10] Schwartz CE, Sprangers MA (2009) Methodological approaches for assessing response shift in longitudinal health-related quality-of-life research. Soc Sci Med 48(11): 1531–1548. 10.1016/s0277-9536(99)00047-7 10400255

[11] Wilson IB (1999) Clinical understanding and clinical implications of response shift. Soc Sci Med 48(11): 1577–1588. 10400258 10.1016/s0277-9536(99)00050-7

[12] Varni JW, Limbers C, Burwinkle TM (2007) Literature review: health-related quality of life measurement in pediatric oncology: hearing the voices of the children. J Pediatr Psychol 32(9): 1151–1163. 17347186 10.1093/jpepsy/jsm008

[13] Fayed N, Schiariti V, Bostan C, Cieza A, Klassen A (2011) Health status and QOL instruments used in childhood cancer research: deciphering conceptual content using World Health Organization definitions. Qual Life Res 2011: 4. 10.1007/s11136-011-9851-5 21293932

[14] Valentini M, Parati G (2009) Variables influencing heart rate. Prog Cardiovasc Dis 52(1): 11–19. 19615488 10.1016/j.pcad.2009.05.004

[15] Thayer JF, Hansen AL, Saus-Rose E, Johnsen BH (2009) Heart rate variability, prefrontal neural function, and cognitive performance: the neurovisceral integration perspective on self-regulation, adaptation, and health. Ann Behav Med 37(2): 141–153. 19424767 10.1007/s12160-009-9101-z

[16] Task Force of the European Society of Cardiology and the North American Society of Pacing and Electrophysiology (2009) Heart rate variability: standards of measurement, physiological interpretation and clinical use. Circulation 93(5): 1043–1065. 8598068

[17] Cysarz D, Linhard M, Edelhauser F, Langler A, Van Leeuwen P, et al (2011) Unexpected Course of Nonlinear Cardiac Interbeat Interval Dynamics during Childhood and Adolescence. PLoS One 6(5): e19400. 21625487 10.1371/journal.pone.0019400 PMC3098842

[18] Patton GC, Viner R (2007) Pubertal transitions in health. Lancet 369(9567): 1130–1139. 17398312 10.1016/S0140-6736(07)60366-3

[19] DiVall SA, Radovick S (2008) Pubertal development and menarche. Ann N Y Acad Sci 1135: 19–28. 18574204 10.1196/annals.1429.026

[20] Nakamura Y, Gang HX, Suzuki T, Sasano H, Rainey WE (2009) Adrenal changes associated with adrenarche. Rev Endocr Metab Disord 10(1): 19–26. 18821019 10.1007/s11154-008-9092-2 PMC3712864

[21] Shirtcliff EA, Dahl RE, Pollak SD (2011) Pubertal development: correspondence between hormonal and physical development. Child Dev 80(2): 327–337. 10.1111/j.1467-8624.2009.01263.x PMC2727719 19466995

[22] Schulz S, Koschke M, Bar KJ, Voss A (2010) The altered complexity of cardiovascular regulation in depressed patients. Physiol Meas 31(3): 303–321. 20086275 10.1088/0967-3334/31/3/003

[23] Rachow T, Berger S, Boettger MK, Schulz S, Guinjoan S, et al (2011) Nonlinear relationship between electrodermal activity and heart rate variability in patients with acute schizophrenia. Psychophysiology 2011(15): 1469–8986. 10.1111/j.1469-8986.2011.01210.x 21496056

[24] Koschke M, Boettger MK, Schulz S, Berger S, Terhaar J, et al (2009) Autonomy of autonomic dysfunction in major depression. Psychosom Med 71(8): 852–860. 19779146 10.1097/PSY.0b013e3181b8bb7a

[25] Brosschot JF, Van Dijk E, Thayer JF (2007) Daily worry is related to low heart rate variability during waking and the subsequent nocturnal sleep period. Int J Psychophysiol 63(1): 39–47. 17020787 10.1016/j.ijpsycho.2006.07.016

[26] Marques AH, Silverman MN, Sternberg EM (2010) Evaluation of stress systems by applying noninvasive methodologies: measurements of neuroimmune biomarkers in the sweat, heart rate variability and salivary cortisol. Neuroimmunomodulation 17(3): 205–208. 20134204 10.1159/000258725 PMC2917732

[27] Bonnemeier H, Richardt G, Potratz J, Wiegand UK, Brandes A, et al (2003) Circadian profile of cardiac autonomic nervous modulation in healthy subjects: differing effects of aging and gender on heart rate variability. J Cardiovasc Electrophysiol 14(8): 791–799. 12890036 10.1046/j.1540-8167.2003.03078.x

[28] Boneva RS, Decker MJ, Maloney EM, Lin JM, Jones JF, et al (2007) Higher heart rate and reduced heart rate variability persist during sleep in chronic fatigue syndrome: a population-based study. Auton Neurosci 137(1–2): 94–101. 17851136 10.1016/j.autneu.2007.08.002

[29] Burton AR, Rahman K, Kadota Y, Lloyd A, Vollmer-Conna U (2010) Reduced heart rate variability predicts poor sleep quality in a case-control study of chronic fatigue syndrome. Exp Brain Res 204(1): 71–78. 20502886 10.1007/s00221-010-2296-1

[30] Bai X, Li J, Zhou L, Li X (2009) Influence of the menstrual cycle on nonlinear properties of heart rate variability in young women. Am J Physiol Heart Circ Physiol 2009 297(2): H765–774. 10.1152/ajpheart.01283.2008 19465541

[31] Chessa M, Butera G, Lanza GA, Bossone E, Delogu A, et al (2002) Role of heart rate variability in the early diagnosis of diabetic autonomic neuropathy in children. Herz 27(8): 785–790. 12574897 10.1007/s00059-002-2340-4

[32] Christ M, Seyffart K, Tillmann HC, Wehling M (1999) Heart rate variability and hormone replacement therapy. Lancet 354(9186): 1303. 10520665 10.1016/S0140-6736(05)76079-7

[33] Charkoudian N, Rabbitts JA (2009) Sympathetic neural mechanisms in human cardiovascular health and disease. Mayo Clin Proc 84(9): 822–830. 19720780 10.4065/84.9.822 PMC2735432

[34] Gilliam FR 3rd, Kaplan AJ, Black J, Chase KJ, Mullin CM (2007) Changes in heart rate variability, quality of life, and activity in cardiac resynchronization therapy patients: results of the HF-HRV registry. Pacing Clin Electrophysiol 30(1): 56–64. 17241316 10.1111/j.1540-8159.2007.00582.x

[35] Kardelen F, Akcurin G, Ertug H, Akcurin S, Bircan I (2006) Heart rate variability and circadian variations in type 1 diabetes mellitus. Pediatr Diabetes 7(1): 45–50. 10.1111/j.1399-543X.2006.00141.x 16489974

[36] Riihimaa PH, Suominen K, Knip M, Tapanainen P, Tolonen U (2002) Cardiovascular autonomic reactivity is decreased in adolescents with Type 1 diabetes. Diabet Med 19(11): 932–938. 12421430 10.1046/j.1464-5491.2002.00816.x

[37] Mormont MC, Waterhouse J, Bleuzen P, Giacchetti S, Jami A, et al (2000) Marked 24-h rest/activity rhythms are associated with better quality of life, better response, and longer survival in patients with metastatic colorectal cancer and good performance status. Clin Cancer Res 6(8): 3038–3045. 10955782

[38] Hathaway DK, Wicks MN, Cashion AK, Cowan PA, Milstead EJ, et al (2000) Posttransplant improvement in heart rate variability correlates with improved quality of life. West J Nurs Res 22(6): 749–768. 11094577 10.1177/01939450022044728

[39] Kummell HC, Van Leeuwen P, Heckmann C, Engelke P, Kesting G, et al (1993) Quality of life and circadian variation of heart rate and heart rate variability in short-term survivors and nonsurvivors after acute myocardial infarction. Clin Cardiol 16(11): 776–782. 8269654 10.1002/clc.4960161106

[40] Buccelletti E, Gilardi E, Scaini E, Galiuto L, Persiani R, et al (2009) Heart rate variability and myocardial infarction: systematic literature review and metanalysis. Eur Rev Med Pharmacol Sci 13(4): 299–307. 19694345

[41] Kemp AH, Quintana DS, Gray MA, Felmingham KL, Brown K, et al (2010) Impact of depression and antidepressant treatment on heart rate variability: a review and meta-analysis. Biol Psychiatry 67(11): 1067–1074. 20138254 10.1016/j.biopsych.2009.12.012

[42] Okano Y, Tochikubo O, Umemura S (2007) Relationship between base blood pressure during sleep and health-related quality of life in healthy adults. J Hum Hypertens 21(2): 135–140. 17096005 10.1038/sj.jhh.1002117

[43] Buchheit M, Simon C, Charloux A, Doutreleau S, Piquard F, et al (2006) Relationship between very high physical activity energy expenditure, heart rate variability and self-estimate of health status in middle-aged individuals. Int J Sports Med 27(9): 697–701. 16944398 10.1055/s-2005-872929

[44] Audette JF, Jin YS, Newcomer R, Stein L, Duncan G, et al (2006) Tai Chi versus brisk walking in elderly women. Age Ageing 35(4): 388–393. 16624847 10.1093/ageing/afl006

[45] Buchheit M, Simon C, Charloux A, Doutreleau S, Piquard F, et al (2005) Heart rate variability and intensity of habitual physical activity in middle-aged persons. Med Sci Sports Exerc 37(9): 1530–1534. 16177605 10.1249/01.mss.0000177556.05081.77

[46] Okano Y, Hirawa N, Matsushita K, Tamura K, Kihara M, et al (2005) Implication of base heart rate in autonomic nervous function, blood pressure and health-related QOL. Clin Exp Hypertens 27(2–3): 169–178. 15835379

[47] Okano Y, Hirawa N, Tochikubo O, Mizushima S, Fukuhara S, et al (2004) Relationships between diurnal blood pressure variation, physical activity, and health-related QOL. Clin Exp Hypertens 26(2): 145–155. 15038625 10.1081/ceh-120028553

[48] Berntson GG, Bigger JT, Eckberg DL, Grossman P, Kaufmann PG, et al (1997) Heart rate variability: Origins, methods, and interpretive caveats. Psychophysiology 34(6): 623–648. 9401419 10.1111/j.1469-8986.1997.tb02140.x

[49] Roach D, Wilson W, Ritchie D, Sheldon R (2004) Dissection of long-range heart rate variability: controlled induction of prognostic measures by activity in the laboratory. J Am Coll Cardiol 43(12): 2271–2277. 15193692 10.1016/j.jacc.2004.01.050

[50] Berntson GG, Cacioppo JT (2004) Heart rate variability: Stress and psychiatric conditions. In: Malik M, Camm AJ (eds.) Dynamic electrocardiography. Futura, New Work 2004 p. 57–64.

[51] Tapanainen J, Thomsen P, Kober L, Torp-Pedersen C, Makikallio T, et al (2002) Fractal analysis of heart rate variability and mortality after an acute myocardial infarction. Am J Cardiol 90(4): 347–352. 12161220 10.1016/s0002-9149(02)02488-8

[52] Pincus SM, Goldberger AL (1994) Physiological time-series analysis: What does regularity quantify? Am J Physiol 266(4): H1643–H1656. 8184944 10.1152/ajpheart.1994.266.4.H1643

[53] Calaminus G, Weinspach S, Teske C, Gobel U (2000) Quality of life in children and adolescents with cancer. First results of an evaluation of 49 patients with the PEDQoL questionnaire. Klin Padiatr 212(4): 211–215. 10994553 10.1055/s-2000-9679

[54] Calaminus G, Weinspach S, Teske C, Gobel U (2007) Quality of survival in children and adolescents after treatment for childhood cancer: the influence of reported late effects on health related quality of life. Klin Padiatr 219(3): 152–157. 17525909 10.1055/s-2007-973846

[55] Kim do H, Kim JA, Choi YS, Kim SH Lee JY, et al (2010) Heart rate variability and length of survival in hospice cancer patients. J Korean Med Sci 25(8): 1140–1145. 20676323 10.3346/jkms.2010.25.8.1140 PMC2908781

[56] Yang AC, Tsai SJ, Yang CH, Kuo CH, Chen TJ, et al (2011) Reduced physiologic complexity is associated with poor sleep in patients with major depression and primary insomnia. J Affect Disord 131(1–3): 179–185. 21195485 10.1016/j.jad.2010.11.030

[57] Spiegelhalder K, Fuchs L, Ladwig J, Kyle SD, Nissen C, et al (2011) Heart rate and heart rate variability in subjectively reported insomnia. J Sleep Res, 20(1 Pt 2): 137–145. 10.1111/j.1365-2869.2010.00863.x 20626615

[58] Marshall SJ, Biddle SJ, Gorely T, Cameron N, Murdey I (2004) Relationships between media use, body fatness and physical activity in children and youth: a meta-analysis. Int J Obes Relat Metab Disord 28(10): 1238–1246. 15314635 10.1038/sj.ijo.0802706

[59] Nagai N, Moritani T (2004) Effect of physical activity on autonomic nervous system function in lean and obese children. Int J Obes Relat Metab Disord 28(1): 27–33. 14710167 10.1038/sj.ijo.0802470

[60] Rodriguez-Colon SM, Bixler EO, Li X, Vgontzas AN, Liao D (2011) Obesity is associated with impaired cardiac autonomic modulation in children. Int J Pediatr Obes 6(2): 128–134. 20919806 10.3109/17477166.2010.490265 PMC3647369

[61] Guizar JM, Ahuatzin R, Amador N, Sanchez G, Romer G (2005) Heart autonomic function in overweight adolescents. Indian Pediatr 42(5): 464–469. 15923693

[62] Griffiths LJ, Parsons TJ, Hill AJ (2010) Self-esteem and quality of life in obese children and adolescents: a systematic review. Int J Pediatr Obes 5(4): 282–304. 20210677 10.3109/17477160903473697

[63] Aziz W, Schlindwein FS, Wailoo M, Biala T, Rocha FC (2012) Heart rate variability analysis of normal and growth restricted children. Clin Auton Res 22(2): 91–97. 22045365 10.1007/s10286-011-0149-z

[64] van Gestel AJ, Kohler M, Steier J, Teschler S, Russi EW, et al (2011) Cardiac autonomic dysfunction and health-related quality of life in patients with chronic obstructive pulmonary disease. Respirology 2011(12): 1440–1843. 10.1111/j.1440-1843.2011.01992.x 21564403

[65] Newton JL, Pairman J, Hallsworth K, Moore S, Plotz T, et al (2011) Physical activity intensity but not sedentary activity is reduced in chronic fatigue syndrome and is associated with autonomic regulation. Qjm 2011: 7. 10.1093/qjmed/hcr029 21382927

[66] von Kanel R, Saner H, Kohls S, Barth J, Znoj H, et al (2009) Relation of heart rate recovery to psychological distress and quality of life in patients with chronic heart failure. Eur J Cardiovasc Prev Rehabil 16(6): 645–650. 19801939 10.1097/HJR.0b013e3283299542

[67] Coelho L, Saraiva S, Guimaraes H, Freitas D, Providencia LA (2001) Autonomic function in chronic liver disease assessed by Heart Rate Variability Study. Rev Port Cardiol 20(1): 25–36. 11291332

[68] Yang TF, Wong TT, Chang KP, Kwan SY, Kuo WY, et al (2001) Power spectrum analysis of heart rate variability in children with epilepsy. Childs Nerv Syst 2001 17(10): 602–606. 10.1007/s003810100505 11685522

[69] Faulkner MS, Hathaway D, Tolley B (2003) Cardiovascular autonomic function in healthy adolescents. Heart Lung 32(1): 10–22. 12571544 10.1067/mhl.2003.6

[70] Faulkner MS, Hathaway DK, Milstead EJ, Burghen GA (2001) Heart rate variability in adolescents and adults with type 1 diabetes. Nurs Res 2001 50(2): 95–104. 10.1097/00006199-200103000-00005 11302298

